# A Role for Voltage-Dependent Anion Channel Vdac1 in Polyglutamine-Mediated Neuronal Cell Death

**DOI:** 10.1371/journal.pone.0001170

**Published:** 2007-11-14

**Authors:** Tanay Ghosh, Neeraj Pandey, Arindam Maitra, Samir K. Brahmachari, Beena Pillai

**Affiliations:** 1 Institute of Genomics and Integrative Biology (IGIB), New Delhi, India; 2 The Centre for Genomic Applications, New Delhi, India; DER Neurogenetics, National Institute of Neurological Disorders and Stroke, United States of America

## Abstract

Expansion of trinucleotide repeats in coding and non-coding regions of genes is associated with sixteen neurodegenerative disorders. However, the molecular effects that lead to neurodegeneration have remained elusive. We have explored the role of transcriptional dysregulation by TATA-box binding protein (TBP) containing an expanded polyglutamine stretch in a mouse neuronal cell culture based model. We find that mouse neuronal cells expressing a variant of human TBP harboring an abnormally expanded polyQ tract not only form intranuclear aggregates, but also show transcription dysregulation of the voltage dependent anion channel, Vdac1, increased cytochrome c release from the mitochondria and upregulation of genes involved in localized neuronal translation. On the other hand, unfolded protein response seemed to be unaffected. Consistent with an increased transcriptional effect, we observe an elevated promoter occupancy by TBP in vivo in TATA containing and TATA-less promoters of differentially expressed genes. Our study suggests a link between transcriptional dysfunction and cell death in trinucleotide repeat mediated neuronal dysfunction through voltage dependent anion channel, Vdac1, which has been recently recognized as a critical determinant of cell death.

## Introduction

Expansion of CAG repeats in coding regions resulting in expanded polyglutamine stretches in proteins is associated with nine neurodegenerative disorders. Though the mechanistic basis of trinuleotide repeat expansion has been studied extensively, the molecular effects that lead to neurodegeneration have remained elusive, in spite of common features of these disorders implying a shared mechanism [reviewed in [1]]. Huntington's disease, the most common of the neurodegenerative disorders caused by the expansion of a CAG repeat in the coding region of the Htt protein has been studied extensively to establish the mechanism of neurodegeneration and for developing therapeutic applications [2]. In many cases, limited knowledge about the normal functions of the protein has prevented studies into the loss/gain of function due to Trinucleotide Repeat Expansion in them.

Spinocerebellar Ataxia 17, is caused by the expansion of a polyglutamine repeat in the poorly conserved, structurally uncharacterized N- terminal half of the human TATA binding protein. The length of the polyglutamine stretch ranges from 29 to 42 in normal individuals whereas in patients they maybe as high as 63 [3]–[5]. The disease is characterized by neurodegeneration in specific regions of the brain at the morphological level and clinical symptoms like gait disturbance, tremor and dementia. TBP a general transcription factor for a majority of eukaryotic promoters, binds to the core promoter element, TATA box (TAT/AAAT) upstream of transcription start site and initiates transcription by allowing assembly of pre-initiation complex [6], [7]. The detection of TBP in intranuclear protein aggregates in brain tissues from Huntington and Alzheimer patients [8] and the association of expanded polyglutamine stretches in TBP with Huntington like symptoms in a Caucasian family [9] have led to the proposal that TBP may play a more general role in a common mechanism for trinucleotide repeat expansion mediated neurodegeneration [8], [10].

Out of a number of mechanisms proposed for neurodegeneration in trinucleotide repeat expansion disorders, transcriptional dysregulation and inappropriate response to unfolded protein accumulation have gained attention in recent years. The strongest evidence for transcriptional dysregulation comes from the observed interaction between the glutamine rich regions of Sp1 with Huntingtin protein harbouring polyglutamine expansion in brain tissue from Huntington patients [11]. Riley and Orr have recently highlighted the importance of transcriptional regulation in polyglutamine disorders and suggested that studying the role of TBP in neurons will help understand how mutations in ubiquitous transcription factors result in disease effects in a restricted set of neurons [12].

Here, we have explored the role of transcriptional dysregulation by expanded polyglutamine stretch containing TBP in SCA17 using a mouse neuronal cell culture based model. Mouse neuronal cells expressing a variant of human TBP harboring 59Q repeats were seen to accumulate intranuclear aggregates whereas a variant harbouring a 16Q stretch did not show any signs of aggregation. Genes involved in localized neuronal translation, cytoplasmic beta-actin (Actb), eukaryotic elongation factor2 (Eef2) and eukaryotic elongation factor 1 (Eef1alpha 1); retrograde transport, p25 subunit of dynactin (Dctn5); survival related gene, Vdac1 and ubiquitin related gene, ubiquitin B (Ubb) were induced in cells with TBP containing expanded polyQ. On the other hand, unfolded protein response seemed to be unaffected. Chromatin Immunoprecipitation studies at a TATA containing and a TATA less promoter from the differentially expressed genes suggested that TBP occupancy was elevated in vivo. Overexpression of the mitochondria and ER associated voltage gated anion channel, Vdac1, has been shown to affect mitochondrial flux and trigger apoptosis in non-neuronal cellular models. In our studies, VDAC1 overexpression lead to elevated cytochrome c release and apoptotic cell death in mouse neuronal cells. We found increased cytochrome c release and apoptotic cell death from the mitochondria in mouse neuronal cells expressing a variant of human TBP harboring abnormally expanded polyQ tract. Our results identify putative link between transcriptional dysregulation and cell death in trinucleotide repeat associated neuronal dysfunction.

## Results

**Figure 1:**
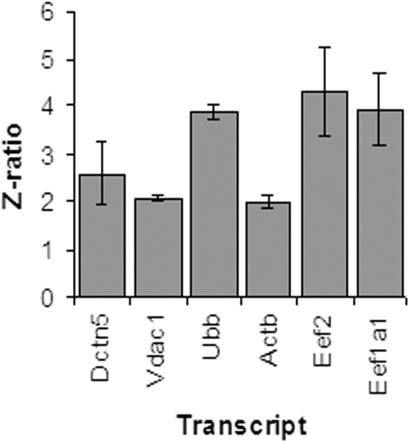
Up-regulation of Dctn5, Vdac1, Ubb, Actb, Eef2 and Eef1a1 in 59QTBP-GFP transfected Neuro-2a cells compared to vector transfected controls observed by cDNA-microarray analysis. X-axis depicts transcript name. Z-ratio of each transcript was plotted on Y axis. Error bar represents mean±SEM of at least two independent microarray experiments.

**Figure 2:**
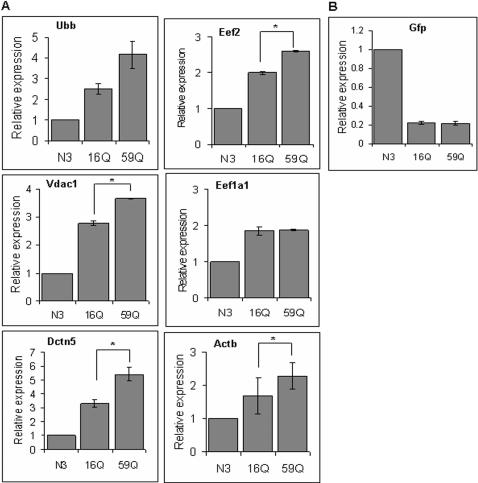
Real time PCR analysis of gene expression. (A) Differential gene expression of Ubb, Vdac1, Dctn5, Eef2, Eef1a1 and Actb in Neuro-2a cell line trasfected with human TBP (hTBP) containing different lengths of polyglutamine. Mouse 18s rRNA was used as an endogenous reference. N3 was used as a calibrator for determining relative expression. (B) Expression levels of reporter (Gfp) in transfected Neuro-2a cells determined by Real time PCR: similar levels of expression of TBPGFP in 16Q and 59Q transfected cells. N3: tranfected construct contains GFP; 16Q, 59Q: transfected construct contains GFP tagged hTBP with CAG repeat length 16 and 59 respectively. Data shown are the mean±SEM of two to four independent experiments. (*, p<0.05, Student's t-test)

**Figure 3:**
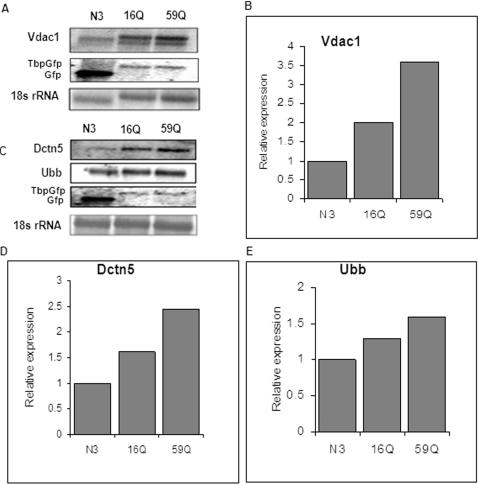
Differential expression of Vdac1 gene, Dctn5 and Ubb in Neuro-2a cells transfected with N3, 16QTBPGFP and 59Q TBPGFP. (A & C) Northern blot using Vdac1 (A), Dctn5 and Ubb as a probe (C), Gfp probe was used to show expression levels of Gfp and TbpGfp in transfected cells (A, C), 18s rRNA was used as a loading control (lower panels in A & C); (B, D & E) The intensity of Vdac1 (B), Dctn5 (D) and Ubb (E) band were quantified and each band intensity was normalised to the band of 18s rRNA. N3 band intensity was used for determining relative expression. N3: tranfected construct contains GFP; 16Q, 59Q: transfected construct contains GFP tagged hTBP with CAG repeat length 16 and 59 respectively.

We used Neuro-2a cells transfected with 16Q and 59Q polyQ containing TBP alleles in fusion with GFP to study the effect of polyQ length in TBP on transcription profile from promoters in their natural genomic context. We substantiated the model by cytological observation of large intranuclear aggregates only in cells transfected with TBP variants carrying expanded polyQ (Fig. S1, Supporting information). A comparison of the microarray based transcription profile of cells transfected with vector and 59Q showed distinct differences. Survival related gene, Voltage dependent anion channel (Vdac1) and ubiquitin related gene, ubiquitin B (Ubb) and genes involved in localized neuronal translation, cytoplasmic beta-actin (Actb), eukaryotic elongation factor2 (Eef2) and eukaryotic elongation factor 1 (Eef1alpha 1); retrograde transport, p25 subunit of dynactin (Dctn5), were amongst the genes that showed significant upregulation in 59Q transfected cells as compared to internally normalized expression levels in vector transfected controls (Fig. 1). The results from microarray experiments were confirmed using real-time PCR analysis (Fig. 2A) and northern blotting (Fig. 3). In comparison with vector transfected controls, 16Q-TBP transfected cells showed marginal elevation of the expression levels of all the genes tested. But the level of induction was however significantly lower than that in 59Q TBP transfected cells in all cases barring Eef1a1. The upregulation of genes in 16Q TBP may be a consequence of higher total levels of functional TBP resulting from expression of the endogenous as well as the transfected gene. The situation in 59Q TBP transfected cells is likely to be totally different due to the apparent aggregation of a significant fraction of the transfected protein (Fig. S1, Supplemental materials). Any indirect effect arising from differential transfection or expression of the transfected TBP in 16Q and 59Q constructs was ruled out by real-time PCR analysis and northern analysis of GFP transcript expressed in fusion with the transfected TBP alleles (Fig. 2B, Fig. 3A–C).

It has been shown that many gene specific transcription factors exert their effects by directly or indirectly interacting with TBP and stabilizing TBP-TATA box interaction at the proximal promoter region [13], [14]. Therefore we studied the effect of polyQ length on TBP occupancy at promoters of differentially expressed genes by chromatin immunoprecipitation(ChIP) using TBP specific N12 antibody. We used anti-TBP antibody rather than the 1C2 antibody against polyQ as 1C2 antibody interacts with polyQ stretches in other transcription factors also and anti-TBP antibody recognizes TBP expressed from the endogenous gene as well as transfected gene. Total TBP localization to promoters seems to be a better index of promoter occupancy since normal and expanded polyQ TBP co-exists in the cell and may form heterodimers.

**Figure 4:**
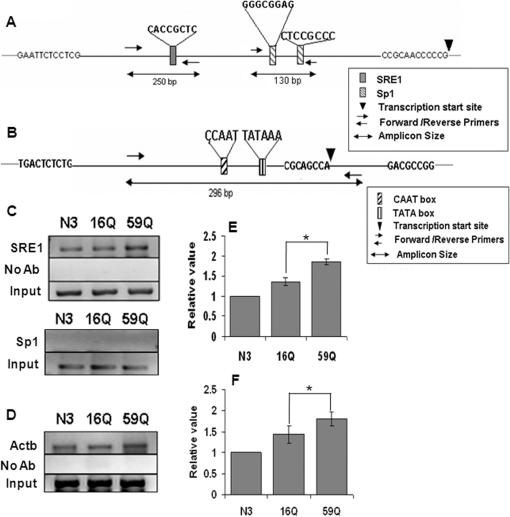
Chromatin immunoprecipitation (ChIP) analysis revealed promoter localization of TBP in transfected Neuro-2a cells. Promoter occupancy of TBP was higher in 59Q TBPGFP transfected cells as compared to vector (N3) and 16Q TBPGFP transfected cells. (A & B) schematic representation of regulatory sites in Vdac1 promoter [predicted (16)] (A) and Actb promoter (B); SRE1: Sterol repressor element 1, Sp1: specificity protein 1. Cells transfected with 16QTBPGFP and 59Q TBPGFP were treated with 1% formaldehyde and chromatin prepared as described earlier [15]. Anti TBP antibody (N-12, Santa cruz) was used for immuno-precipitation. PCR was performed from immunoprecipitates, total input chromatin (Input). Samples treated without antibody (No Ab) did not show any product after PCR. PCR product for SRE1 (upper), Sp1 (lower) (C) and Actb promoter (D) were resolved on agarose gel. (E & F) intensity of band for SRE1 (E) and Actb promoter (F) were quantified and normalized to input band. Data represented relative to the N3 transfected control. Data shown are mean±SEM of three independent experiments performed. (*, p<0.01, Student's t-test).

Promoter occupancy of TBP was higher in 59Q TBP transfected cells compared to N3 and 16QTBP transfected cells at the Vdac1 and Actb promoters (Fig. 4). The Vdac1 gene was predicted to be under the control of a TATA-less promoter [15]. The predicted Sp1 site in the Vdac1 gene was of special interest since aberrant Sp1 mediated interaction has earlier been implicated in Huntington's disease mechanism [11]. TBP localization could not be demonstrated in spite of repeated attempts at amplifying the region spanning the predicted Sp1 site (Fig. 4C, lower). On the other hand, TBP occupancy at the sterol repressor element 1 (SRE1) site, further upstream was enhanced in the presence of expanded polyQ variants (Fig. 4C, upper). We examined the downstream cellular effects of the upregulation of Ubb and Vdac1 to correlate the changes in gene expression to neurodegeneration.

**Figure 5:**
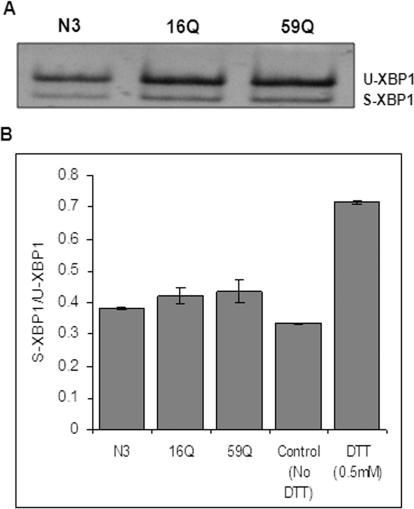
Poly Q expanded TBP transfected cells do not show ER stress. ER stress was detected by reverse transcription (RT) reaction followed by PCR amplification of spliced (S) and unspliced Xbp1 (U). PCR products were resolved in 5% polyacrylamide gel, stained with vista green. (A) Unspliced and spliced Xbp1 PCR products detected in vector (N3), 16QTBPGFP and 59QTBPGFP transfected Neuro-2a cells (B) Vista green stained bands were quantified and ratio (spliced/unspliced) was calculated. Higher ratio in DTT treated positive control indicates ER stress. 59QTBPGFP and 16QTBPGFP transfected cells were showing similar ratio as observed in vector (N3) transfected control. Data represent mean±SEM of two independent experiments.

The cell responds to aggregated protein accumulation in two ways: initially a corrective mechanism is adopted through the unfolded protein response/ ER stress pathway, which induces chaperones and provides an environment for correct folding of the proteins and finally, ubiquitinylation and destruction of the malformed proteins are attempted. Ubb was amongst the genes with increased expression in cells expressing expanded polyQ containing TBP. We analyzed the Unfolded Protein Response pathway using xbp1-splicing assay as an indicator [16]. The ratio of the spliced and unspliced variants of xbp1 showed no alteration in cells expressing the different polyQ TBP alleles (Fig. 5).

**Figure 6:**
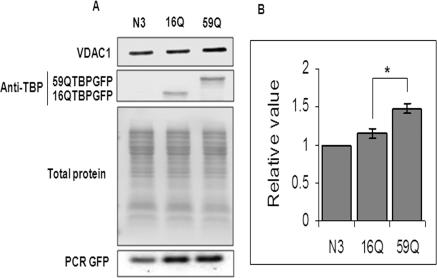
Expression of VDAC1 protein in Neuro-2a cells transfected with vector (N3), 16QTBPGFP and 59QTBPGFP. (A) Western blotting with VDAC1 specific antibody (N-18, Santa cruz) [upper panel], TBP specific antibody (N-12, Santa cruz) [middle panel]. Equal sample loading was verified by staining the blot with Ponceau S. DNA was isolated from transfected cells (see materials & methods) and equivalent amount of plasmid transfection was checked by PCR amplification using GFP specific primers (Table 1) (B) Immunoreactive band intensity was quantified and plotted. Data presented relative to the vector (N3) transfected control. Data shown are mean±SEM of three independent experiments performed. (*, p<0.05, Student's t-test)

**Figure 7:**
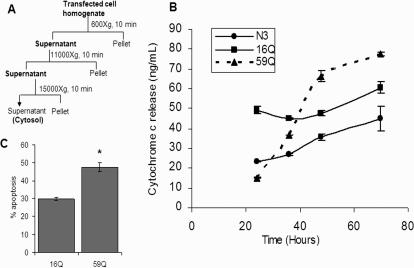
Quantitative analysis of cytochrome c release into the cytosol of transfected Neuro-2a cells. (A) Scheme for isolation of cytosol from cultured cells; (B) Time dependent release of cytochrome c in the cytosol was measured by using a solid phase ELISA assay. Data represents mean±SEM (n = 2). (C) Apoptosis in 16Q TBPGFP and 59Q TBPGFP transfected cells were analyzed by counting annexinV-PE positive cells in a flow cytometer. Data represents mean±SEM of four independent experiments performed. (*, p<0.01, Student's t-test).

Recently, the voltage dependent anion channel, Vdac1, has been shown to localize to both the mitochondrial outer membrane and the cytoplasmic membrane [17]. A change in the expression level of Vdac1 has recently been shown to be a critical determinant of cell death in embryonic kidney cells [18]. Vdac1 plays an important role in transport of ATP, calcium ions and other metabolites across the mitochondrial membrane [19]. Calcium induced permeability transition and cytochrome c release from the mitochondria is enhanced in cellular models expressing the mutant huntingtin protein [20]. Further, Ruan et al., observed that mutant huntingtin was unable to release cytochrome c in the cytosol and striatal cells undergo non-apoptotic death [21]. We first confirmed that the transcriptional upregulation of Vdac1 was associated with a similar increase in the expression level of the protein. As shown in Fig 6, a specific antibody against VDAC1 was used to monitor the level of VDAC1 protein in cells transfected with a vector control, 16Q TBP or 59Q TBP. VDAC1 protein levels were induced by 30 percent in 59Q TBP containing cells. Further, we studied cytochrome c release from the mitochondria following increase in VDAC1 expression. The scheme for subcellular fractionation is presented in Fig 7A. Cytochrome c release in the cytosolic fraction was monitored by ELISA. The cells expressing 59Q TBP showed a gradual increase in cytochrome c release resulting in substantially higher Cyt c levels at time points beyond 36 hours compared to cells which express the vector alone (Fig. 7B), in agreement with increased expression of VDAC1. We further monitored apoptotic cell death using flow cytometry of AnnexinV positive cells. Neuro-2a cells harbouring polyglutamine expanded TBP showed 48% apoptotic cells while control cells showed less than 30% apoptotic cells (Fig. 7C).

**Table 1:** Mouse specific primer sequences and amplicon sizes used in real time RT-PCR.

Gene	Sequence (5′-3′)	Amplicon size (bp)
Dctn5	F: CGAGGAAGACTGTGTGGTCA	192
	R: AGCTCCCCTGAGAAGAGTCC	
Actb	F: CTAAGGCCAACCGTGAAAAG	126
	R: CCATCACAATGCCTGTGGTA	
Vdac1	F: AATGACGGGACAGAGTTTGG	231
	R: AATGACGGGACAGAGTTTGG	
Eef1a1	F: CTTCTCTGACTACCCTCCAC	60
	R: CCACAGCTTTGATGACACCC	
Eef2	F: TGAGCAAGTGGTGGGTGG	134
	R: TGTTGGATCGCAGATCAGCGG	
Ubb	F: TCTTTCTGTGAGGGTGTTTCG	131
	R: GTCACTGGGCTCCACCTCTA	
18s rRNA	F: CTTTCGAGGCCCTGTAATTG	63
	R: CCTCCAATGGATCCTCGTTA	
GFP	F: CTACAACAGCCACAACGTC	127
	R: GGTGTTCTGCTGGTAGTGGT	

The table lists the sequences (5′ to 3′) of the forward and reverse primers used in real time PCR reaction to amplify various gene transcripts. The size of the amplicon is given as base pairs (bp). The designation “F” indicates forward and “R” indicates reverse.

**Figure 8:**
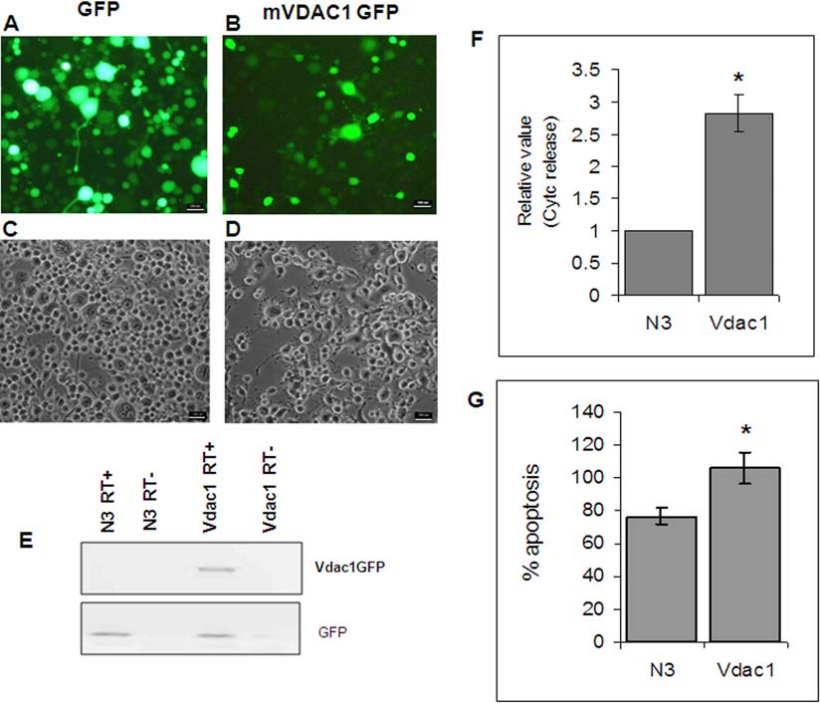
Over-expression of murine Vdac1 (mVdac1) in Neuro-2a cells induces apoptosis. (A–D) Neuro-2a cells transfected with pEGFPN3 vector alone (N3) (A & C) and mVdac1GFP (B & D) [A & B, Fluorescent field; C & D, Bright field view under 10× objective in an epifluorescence microscope (Nikon)]. (Scale bar,100 um). (E) Expression of mVdac1Gfp in Neuro-2a cells was confirmed by Reverse transcription followed by PCR (RT-PCR) amplification from cDNA using a Vdac1 specific forward primer and GFP specific reverse primer (Upper panel). Expression of GFP was checked in N3 and mVdac1GFP transfected cells by RT-PCR using GFP specific forward and reverse primers. RT+: with reverse transcriptase, RT-: without reverse transcriptase. (F) Release of cytochrome c into cytosol in mVdac1 transfected cells was determined by using a solid phase ELISA method 65H after transfection. Data is presented relative to vector transfected control (N3). Data represents mean±SEM of two independent experiments each in replicate. (*, p<0.01, Student's t-test) (G) Flow cytometric analysis of apoptosis in mVdac1GFP and N3 transfected cells (65H after transfection) was determined by counting annexinV-PE positive cells. Data represents mean±SEM of two independent experiments, each in replicate. (*, p<0.05, Student's t-test).

Previous studies have shown that overexpression of VDAC1 can lead to apoptosis in non-neuronal cells. We confirmed a similar effect of VDAC1 in neuronal cells by cloning and overexpressing mouse VDAC1 (Fig. 8A–E). Cytochrome c release was three fold higher in VDAC1 overexpressing cells (Fig. 8F). Consistent with cytochrome c release, flow cytometric analysis of AnnexinV-Phycoerythrin (AnnexinV-PE) positive cells revealed that VDAC1 overexpression induced apoptosis (Fig. 8G).

## Discussion

In this study we show that alleles of TBP harbouring abnormally expanded polyQ tracts not only form intranuclear aggregates in cultured mouse neuronal cells, but also result in overexpression of the voltage dependent anion channel, Vdac1 at both the RNA and protein level. VDAC can associate with small ions, control the passage of key adenine nucleotides, interact with other proteins like cellular kinases and regulate mitochondrial permeability [17], [19]. VDAC localizes to the outer mitochondrial membrane and associates with the Adenine nucleotide translocator in the inner mitochondrial membrane to form the permeability transition pore through which cytochrome c is released in apoptotic cells. Pro-apoptotic members of Bcl2 family of proteins like Bax and Bak are seen to promote Cytc release by binding to and modulating Vdac action [22]. We independently confirmed that Vdac1 overexpression in mouse neuronal cells results in higher cytochrome c release and apoptosis, as has been reported for non-neuronal cells previously [23]. In our studies the cells expressing 16Q TBP showed higher constant levels of cytosolic Cyt c compared to cells which express the vector alone. 59Q TBP cells, however, showed a significant difference in the cytochrome c release pattern. At early time points, cytosolic Cyt c levels are comparable to controls but with time they steadily rise and saturate at levels 30% higher than in 16Q TBP cells by 70 hours. Concurrently, 59Q TBP expressing cells showed a 20% increase in number of apoptotic cells.

Besides, SCA17, caused by the expansion of polyQ tract in the TATA binding protein itself, normal TBP is also found in intra nuclear protein aggregates formed in other neurodegenerative diseases, notably, huntington's disease [8]. Degeneration of cells in specific regions of the brain is a well known feature of polyQ mediated neurodegenerative disorders. Vdac1 overexpression, independently implicated in triggering cell death was one of the effects of polyglutamine expansion in TBP, identified in our study. Our results suggest a previously unknown mechanistic basis linking transcriptional dysregulation to cell death in neurodegeneration through TBP mediated alteration of Vdac1 expression.

The promoter of the Vdac1 is predicted to be devoid of a TATA box [15]. TATA less promoters depend on TBP participation in the pre-initiation complex by localizing it to the promoter through other DNA binding proteins. Even at TATA box containing promoters, TBP binds inefficiently in the presence of chromatin and requires gene specific trans-activators [13]. We find that TBP occupancy at the promoter of upregulated genes was elevated irrespective of the presence of the TATA box. Expansion of polyQ and formation of TBP containing protein aggregates suggest that TBP can cause enhanced expression of a downstream gene by titrating away a gene specific repressor. Alternatively, polyQ containing TBP can form heterodimers in solution with the endogenous functional TBP and alter its availability at various promoters. The structural or functional role of polyQ stretch within TBP has not been studied. The yeast homolog of TBP does not have the N-terminal part harboring the polyQ stretch while a gradual increase in the length of the polyQ stretch during evolution is observed. The conserved C-terminal domain of TBP is sufficient for binding to the TATA box and can be co-crystalized with it [24]. Using an artificially tethered TBP with promoter-reporter fusions and at the genome-wide level it has been shown that many gene specific transcription factors exert their effects by directly or indirectly interacting with TBP and stabilizing TBP-TATA box interaction at the proximal promoter region [13], [14]. The interaction of expanded polyQ containing TBP with other factors in the eukaryotic transcription machinery will provide a more detailed picture of the transcriptional dysregulation.

VDAC action is known to be modulated by pharmacological agents. Besides synthetic and naturally occurring polyanions like spermine which increase the voltage dependence of VDAC channel, ruthenium red can reduce the voltage dependence of VDAC channel [23]. The potent anti-depressant, Prozac (Fluoxetine) has earlier been shown to prevent apoptosis and enhance cell proliferation in the dentate gyrus. Fluoxetine has been shown to interact with the VDAC channel and modify its voltage dependence and conductance and in certain cell types can provide protection from apoptosis [25]. TBP, being a general transcription factor is likely to have diverse, direct and indirect effects on gene expression and consequently in cellular function. These effects are unlikely to be mediated solely through Vdac1. Further investigations using chemical inhibitors or gene knock down strategies to negate the elevated Vdac1 transcript level can delineate the contribution of Vdac1 to the phenotypic effects of polyglutamine expansion in TATA binding protein.

## Materials and Methods

### Generation of GFP fusion clones

The original human TBP construct containing the human TBP cloned in NdeI-BamHI site of pET3b vector (Novagen) has been previously described [26]. The glutamine repeat containing region of TBP was amplified using following primers:

5′-AAGAAGGACATATGGATCAGAACAACAGCC-3′

5′-TGCCACTGCCAGCTGTTGCTGCTG-3′ which respectively introduced NdeI and PvuII restriction endonuclease site in the PCR product I. The flanking region 3′ to the glutamine repeat of the TBP was amplified using following primers:

5′-CAGCAGCAGCAGCAACAGGCAGTGGCA-3′

5′-GCTAGTTATTGCTCAGCGGTGGCAGCAGC-3′ resulting in PCR product II.

The NdeI and PvuII digested PCR I product, was gel purified. PCR product II was end filled by using Vent polymerase, digested with BamHI and gel-purified. These processed PCR products i.e., I and II were cloned into BamHI-NdeI digested pET-15b vector in a three-fragment ligation. However, during the process of cloning, clones containing 16 and 59 glutamine repeats were obtained. The clones were sequenced to confirm the presence of continuous glutamine repeats. The clones were designated as p-15b16QTBP, and p-15b59QTBP.

Further, human TBP gene was amplified from the p-15b16QTBP and p-15b59QTBP for re-cloning into pEGFPN3 vector (Clontech). The following primers introduced XhoI and BamHI site, respectively, in the PCR product:

5′-GGAGATCTCGAGATGGATCAGAACAAC-3′and

5′-GCAGCCGGATCCCGTCGTCTTCCT-3′

Underlined sequences in the primers mentioned above represent the sites for restriction endonucleases. Proof reading KOD polymerase (Toyobo, Osaka, Japan) was used in the PCR reaction and the resultant PCR product was digested with BamHI and XhoI. The gel purified PCR product was cloned in BamHI-XhoI site of pEGFP-N3 (Clontech) vector, in frame with green fluorescent protein (GFP). The pEGFP-N3 vector and constructs were designated as N3, 16Q, and 59Q respectively in this paper.

### Cloning of mVdac1

The coding sequence of murine Vdac1 (mVdac1) (gene bank id NM_011694.3) was amplified from cDNA synthesized from N2a cells. The following primers introduced BglII and KpnI site, respectively, in the PCR product (restriction sequence underlined):

5′-AAAAGATCTATGGCCGTGCCTCCC-3′and

5′AAAGGTACCTGCTTGAAATTCCAGTCC 3′

The gel purified PCR product was cloned in BglII-KpnI site of pEGFP-N3 (Clontech) vector, in frame with green fluorescent protein (GFP) and confirmed by sequencing.

Expression of mVdac1GFP in transfected N2a cells was confirmed by reverse transcription followed by PCR (RT-PCR). The following primers were used for amplification:

5′-AATGACGGGACAGAGTTTGG-3′ (Specific for Vdac1)

5′-GGTGTTCTGCTGGTAGTGGT-3′ (Specific for GFP)

Expected product size was confirmed by resolving the PCR product on a 1% agarose gel.

### Cell culture and transfection

Murine neuroblastoma cells (ATCC number CCL-131; Neuro-2a or N2a) were maintained in Minimum essential medium (MEM) (GIBCO-BRL) supplemented with 10% fetal calf serum [Biological Industries, Israel], 2 mM L-glutamine [Sigma], 1mM sodium pyruvate [Sigma] and antibiotic-antimycotic solution (100× stock) [Sigma] at 37°C humidified incubator with 5% CO_2_. Approximately 3×10^5^ cells were seeded in each well of 6 well plate (Axygen) 24 h prior to transient transfection when 50–80% confluency was reached. Cells were washed once with Opti-MEM (GIBCO-BRL) and maintained in 1 ml Opti-MEM. 1 µg of transfection quality DNA (Endo free plasmid maxi kit; Qiagen) and 3 µl of fugene 6 transfection reagent (Roche) were used for preparation of transfection complex. Transfection of N3, 16Q and 59Q was performed following the protocol from the manufacturer. After 60 h of incubation cells were observed in an inverted epifluorescence microscope (TE200-U, NIKON) under 10× objective. Transfection has also been performed by using Amaxa neucleofection technology. Transfection efficiency was found to be about 45 to 50% and used for normalization.

### Confocal analysis

Described in detail in Text S1.

### RNA isolation and purification

After 60 H of transfection total RNA was isolated from the cells using TRIzol reagent (GIBCO-BRL) according to the manufacturer's instruction. The RNA pellets were washed with 70% ethanol, centrifuged and dried. Pellets were re-suspended into 30 µl of DEPC treated water followed by the addition of 10× reaction buffer and 2 U of RNAse free DNase I (Fermentas) in a total volume of 45 µl. Samples were incubated at 37°C for 30 minutes. Then the RNA was cleaned using RNeasy Mini Kit (Qiagen) following the protocol by the manufacturer. RNA concentration and purity was determined by measuring optical density at 260 nm and 280 nm using a spectrophotometer (Eppendorf) and running the RNA samples on a 1.5% agarose formaldehyde gel.

### Reverse transcription (RT) and real time polymerase chain reaction (PCR)

cDNA was generated from the total RNA samples by using random hexamer (New England biolabs Inc, NEB) and M-MuLV reverse transcriptase (NEB) at 42°C for 1 h. Real time PCR was performed using SYBR green master mix (Applied Biosystems, ABI) in ABI 7500 real time PCR instrument. The forward and reverse primers are found in Table 1. The real time PCR program consisted of activation of uracil-N-glycosylase (UNG) at 50°C for 2 min., then 95°C for 10 min to inactivate UNG and activate ampli Taq Gold DNA polymerase, followed by 40 cycles of denaturation at 95°C for 15 sec, annealing at 55°C for 30 sec and extension at 60°C for 1 min. The products were analyzed on 5% polyacrylamide gel to confirm the appropriate product size.

### Data analysis

Relative quantitation of gene expression was carried out using the mathematical expression described recently [27]. PCR efficiency of target gene and endogenous control gene has been determined from the slope of the respective standard curve.

### Microarray studies and data analysis

Total RNA was isolated from N3 and 59Q transfected N2a cells as mentioned above. Total RNA was labeled by Micromax NEN TSA labeling system (Perkin-Elmer Life Sciences, USA) according to manufacturer instruction. Labeled total RNA was hybridized onto mouse cDNA array (Microarray centre, University Health Network, Ontario, Canada). Six different replicates were performed from the cultured N2a cells in different batches. The slides were scanned using an Axon scanner and data were acquired and analysed using Genepixpro. Data from 59Q transfected (treated) and N3 transfected (control) samples were normalized using Z score transformation method described by Cheadle et al., [28]. Z ratio value ±1.96 was considered significant (p<0.05) [28]. Microarray data has been submitted to GEO (Accession no. GSE5807).

### Northern analysis

Total RNA was transferred to the nylon membrane after separation on a 1.5% agarose formaldehyde gel. Subsequently, radioactively labeled probe prepared from purified PCR products of Vdac1, Dctn5, Ubb and Gfp using NEBlot^TM^ kit (NEB) according to manufacturer instruction. Hybridized probe signal was detected by phosphorimager (Fujifilm FLA 2000IR) and intensity was quantified using MultiGauge software

### Chromatin immunoprecipitation (ChIP)

Chomatin immunoprecipitation reaction was performed using standard protocol [29]. 2 µg anti TBP antibody (N-12∶sc-204; Santa cruz Biotechnology Inc.) was used for immunoprecipitation. Precipitated DNA samples were analyzed by PCR (35–38 cycles). The following forward and reverse primers were used for each gene promoter respectively:

Vdac1 SRE1:

5′-GGGAGAGTTTAATTTGCAACTGACT-3′

5′-CTGGAAGCATTTGGGAAGAG-3′

Vdac1 Sp1:

5′-GAGACTGGTCTGGGCGCTGTC-3′

5′-TGGGAGCGCAGCGAACGGGCC-3′

Actb:

5′-CCATCGCCAAAACTCTTCAT-3′

5′-AAGGAGCTGCAAAGAAGCTG-3′

PCR products were resolved on a 2% agarose gel. Ethidium bromide stained bands were quantified using Quantity one software (BioRad). Band intensity was normalized to the input band.

### PCR-based assay for Xbp1 splicing

cDNA synthesized from total RNA isolated from transfected cells was used as template for PCR. Primers and PCR conditions were used as described by Marciniak et al. [30]. 480 bp product obtained from unspliced Xbp1 while spliced Xbp1 gave 454 bp product. PCR products were separated on 5% polyacrylamide gel and stained with Vista green (Amersham Biosciences, UK). Fluoroimage was captured by gel doc system (BioRad Laboratories, USA) and intensity was quantified using quantity one v 4.1.1 software.

### Western analysis

Neuro-2a cells were transfected with vector alone (N3), 16QTBPGFP and 59QTBPGFP using either fugene6 or Amaxa nucleaofection device. Transfected cells grown on 6-well plate were scrapped after 60–65 h of incubation. Cells were lysed using 1× SDS sample buffer (50 mM Tris-Cl, pH 6.8, 100 mM 2-mercaptoethanol, 2% (w/v) SDS, 10% glycerol) and incubated at 55°C for 1 h, then boiled for 5 min. Total protein was estimated using BCA protein estimation kit (Sigma). Bromo-phenol blue was added to each sample and equal amount of protein was separated on 12% SDS-polyacrylamide gel. The separated protein was transferred to a nitrocellulose membrane and stained with Ponceau S to check equal loading. Membrane was blocked in a solution of 137 mM NaCl, 3 mM KCl, 25 mM Tris-HCl, 0.1% Tween 20, pH 7.4 containing 5% (w/v) skimmed milk for overnight at 4°C. VDAC1 specific polyclonal antibody (N-18: sc-8828) (1∶500 dilution) was added and incubated for 2 h at room temperature, washed and then incubated with alkaline phosphatase (AP) conjugated secondary antibody (1∶3000). For TBP specific western TBP specific polyclonal antibody (N-12∶sc-204) (1∶500 dilution) was used. Immunoreactive bands were detected using BCIP/NBT solution. Western blot was scanned and band intensity was quantified by using Quantity one software (BioRad). Transfected N2a cells were lysed by using a lysis buffer (50 mM Tris-Cl pH 8.1, 10 mM EDTA, 1% SDS), boiled for 10 minutes for complete lysis, and then spun at 5000 rpm to remove debris. DNA has been precipitated from the collected supernatant by using 5 M Nacl to a final concentration 0.3 M and kept for overnight at −20°C. DNA was pelleted down, washed with 70% ethanol and dissolved in TE. PCR amplification from that template has been performed by using GFP specific primers (Table 1); run on a 2% EtBr stained agarose gel to resolve the product.

### Measurement of cytochrome c release

Neuro-2a cells were transfected using Amaxa nucleaofection device following manufacturer protocol. Transfected cells were grown on 6 well plates in Opti-MEM (GIBCO-BRL). Cells were scraped after 24 hr, 36 hr, 48 hr and 70 hr of incubation, washed in PBS, washed in 1× extraction buffer A (10 mM HEPES, pH 7.5, 200 mM manitol, 70 mM sucrose and 1 mM EGTA)[sigma] and re-suspended in the same buffer containing 2 mg ml^−1^ bovine serum albumin (Sigma). After 1 h incubation on ice, cells were lysed by a B-type Dounce homogenizer with 30–35 strokes. Homogenates were centrifuged at 4°C in the subsequent steps to remove nuclei, debris and mitochondria (Fig. 6A). Supernatant was re-centrifuged at 15,000× g at 4°C to get cytosolic fraction. To rule out mitochondrial contamination in the cytosolic fraction a colorimetric assay was performed using cytochrome c oxidase assay kit (Sigma) following manufacturer protocol. Protein estimation in the cytosolic fraction was performed by using BCA protein estimation kit (Sigma). Equal amount of protein from cytosolic fraction was used from N3, 16Q and 59Q transfected samples and cytochrome c concentration was quantitatively examined by a solid phase ELISA kit (Quantikine ® Rat/Mouse, R & D systems, Minneapolis, MN, USA) according to the manufacturer's instructions.

Similar method was followed for Cyt c release assay in case of mVdac1GFP transfected cells. Assay was performed 65 H after transfection.

### Flow cytometry analysis

Untransfected and transfected N2a cells grown on 6 well plate were harvested by trypsinization/scrapping. Untransfected cells were used as a negative control. Cells were stained with Annexin V-PE and 7-AAD by using Nexin^TM^ kit (Guava technologies) following manufacturer's protocol. Data acquisition and analysis were performed by using GuavaEasyCyte flow cytometer and Guava Nexin cytosoft software (v3.6.1). Transfection efficiency has been calculated by counting GFP positive cells in the above mentioned flow cytometer using Guava Express plus software (v3.6.1). For data analysis total annexin V-PE positive cells were normalized to transfection efficiency.

## Supporting Information

Figure S1 Confocal analysis of expressed TBP-GFP fusion protein (green) in the nucleus of transfected Neuro-2a cell line. Nuclei were stained with Propidium Iodide (red fluorescence). 16QTBP-GFP transfected Neuro-2a cells showed diffused localization to the nucleus (A); multiple large intranuclear aggregates were observed in cells expressing 59QTBP-GFP fusion proteins (B). Aggregates are indicated by arrow.
(5.15 MB TIF)

Text S1 (0.02 MB DOC)
