# Supplementary figures and images for: A Role for Voltage-Dependent Anion Channel Vdac1 in Polyglutamine-Mediated Neuronal Cell Death
Source: PLoS One. 2007 Nov 14;2(11):e1170. doi: 10.1371/journal.pone.0001170 (PMC2064964; doi:10.1371/journal.pone.0001170)

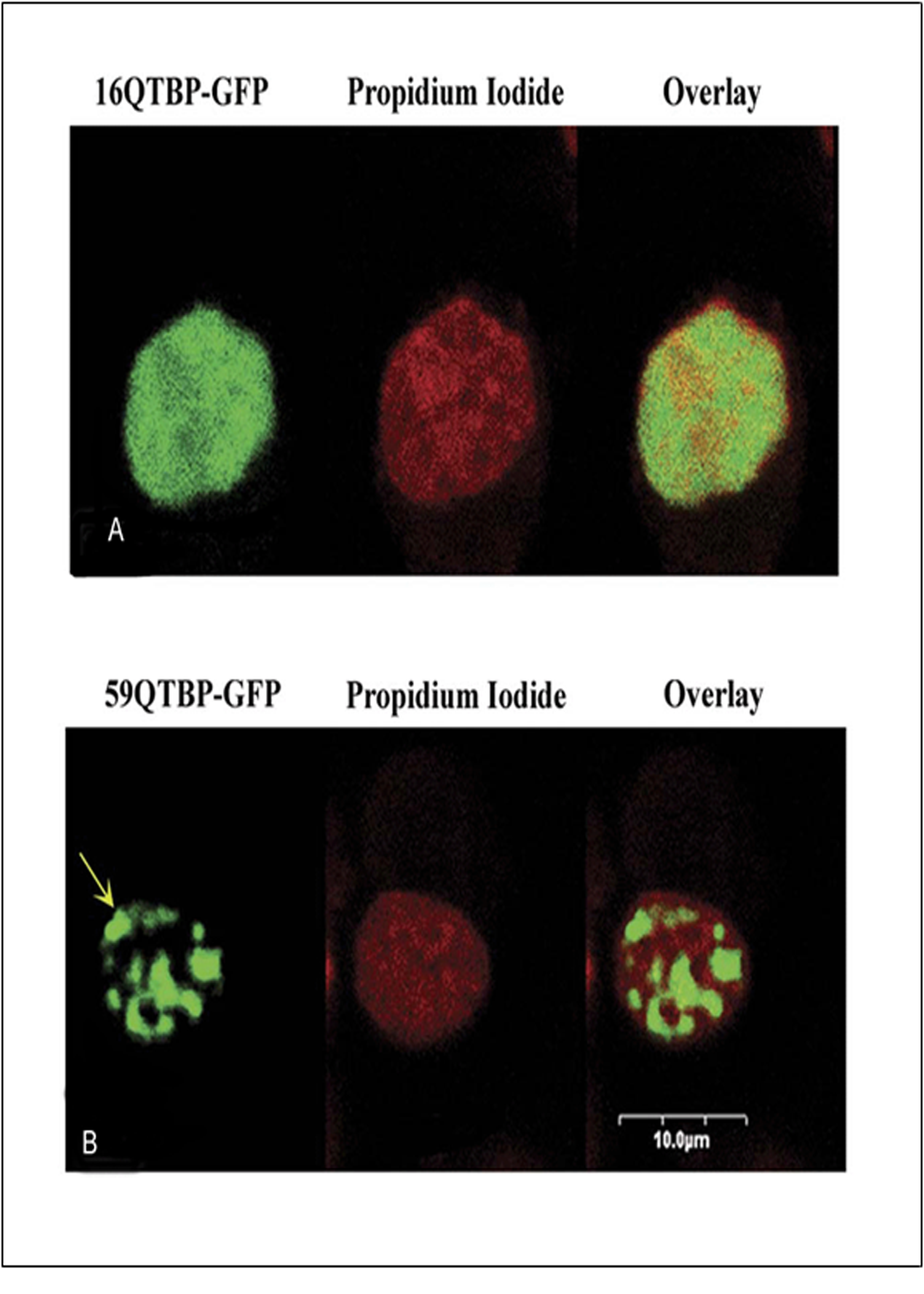

Supplement: Figure S1 — Confocal analysis of expressed TBP-GFP fusion protein (green) in the nucleus of transfected Neuro-2a cell line. Nuclei were stained with Propidium Iodide (red fluorescence). 16QTBP-GFP transfected Neuro-2a cells showed diffused localization to the nucleus (A); multiple large intranuclear aggregates were observed in cells expressing 59QTBP-GFP fusion proteins (B). Aggregates are indicated by arrow. (5.15 MB TIF) [file pone.0001170.s001.tif]
