# Supplementary material for: A Role for Voltage-Dependent Anion Channel Vdac1 in Polyglutamine-Mediated Neuronal Cell Death
Source: PLoS One. 2007 Nov 14;2(11):e1170. doi: 10.1371/journal.pone.0001170 (PMC2064964; doi:10.1371/journal.pone.0001170)
Supplement: Text S1 — (0.02 MB DOC) [file pone.0001170.s002.doc]

**Supporting information:**

**Files in this data supplement**

Supporting Figure S1.

Supporting Text.

GEO accession number of Microarray data: GSE 5807

**Supporting figure legend**

Figure S1: Confocal analysis of expressed TBP-GFP fusion protein (green) in the nucleus of transfected Neuro-2a cell line. Nuclei were stained with Propidium Iodide (red fluorescence). 16QTBP-GFP transfected Neuro-2a cells showed diffused localization to the nucleus (A); multiple large intranuclear aggregates were observed in cells expressing 59QTBP-GFP fusion proteins (B). Aggregates are indicated by arrow.

**Supporting text**

**Confocal analysis:** Cells were grown in chamber slides. Two days after transfection cells were washed twice with PBS, fixed with 4% paraformaldehyde in PBS for 30 mins. Nuclei were stained with Propidium iodide (1µg/ml). Samples were observed using a confocal microscope (Fluoview; Olympus, Tokyo, Japan) and digital images were assembled using Adobe Photoshop.
